# Neuroprotective potential of *Cannabis sativa*-based oils in *Caenorhabditis elegans*

**DOI:** 10.1038/s41598-022-19598-3

**Published:** 2022-09-13

**Authors:** Ana Paula Vanin, Wagner Antonio Tamagno, Carla Alves, Letícia Mesacasa, Luciani Figueiredo Santin, Nathália Tafarel Sutorillo, Denise Bilibio, Caroline Müller, Leandro Galon, Rosilene Rodrigues Kaizer

**Affiliations:** 1 Universidade Federal da Fronteira Sul, Erechim, RS 99.700-000 Brazil; 2 Universidade Federal de Santa Maria, Santa Maria, RS 97.105-900 Brazil; 3 Instituto Federal de Educação, Ciência e Tecnologia do Rio Grande do Sul, Sertão, RS 99.170-000 Brazil; 4 Universidade de Passo Fundo, Passo Fundo, RS 99.052-900 Brazil; 5 Universidade de Santa Cruz do Sul, Santa Cruz do Sul, RS 96.815-900 Brazil

**Keywords:** Drug screening, Enzymes, Neurochemistry, Neuroscience

## Abstract

Substances from the *Cannabis sativa* species, especially cannabidiol (CBD) and Delta-9-tetrahydrocannabinol (Δ9-THC), have attracted medical attention in recent years. The actions of these two main cannabinoids modulate the cholinergic nervous system (CholNS) involving development, synaptic plasticity, and response to endogenous and environmental damage, as a characteristic of many neurodegenerative diseases. The dynamics of these diseases are mediated by specific neurotransmitters, such as the GABAergic nervous system (GNS) and the CholNS. The nematode *Caenorhabditis elegans* is an important experimental model, which has different neurotransmitter systems that coordinate its behavior and has a transgene strain that encodes the human β-amyloid 1–42 peptide in body wall muscle, one of the main proteins involved in Alzheimer´s disease. Therefore, the objective of this study was to evaluate the protective potential of terpenoids found in *C. sativa* in the GNS and CholNS of *C. elegans*. The effect of two *C. sativa* oils with variations in CBD and THC concentrations on acetylcholinesterase (AChE) activity, lipid peroxidation, and behavior of *C. elegans* was evaluated. *C. sativa* oils were efficient in increasing pharyngeal pumping rate and reducing defecation cycle, AChE activity, and ROS levels in N2 strains. In the *muscle:Abeta1-42* strain, mainly when using CBD oil, worm movement, body bends, and pharyngeal pumping were increased, with a reduced AChE activity. Consequently, greater investments in scientific research are needed, in addition to breaking the taboo on the use of the *C. sativa* plant as an alternative for medicinal use, especially in neurodegenerative diseases, which have already shown positive initial results.

## Introduction

The *Cannabis sativa* plant has hundreds of chemical compounds produced by secondary metabolism, including cannabinoids, terpenes, and phenolic compounds, each with potential biological properties^1^. Among the cannabinoids, delta-9-tetrahydrocannabinol (Δ9-THC) and cannabidiol (CBD) stand out as the two main ones^2^.

THC is well known for its psychoactive properties, while CBD is a non-psychostimulant cannabinoid^3^. These cannabinoids modulate the cholinergic nervous system (CholNS) involving development, synaptic plasticity, and response to endogenous and environmental damage, as a characteristic of many neurodegenerative diseases^4^. Through the interaction between cannabinoids and their receptors, an anti-inflammatory response is generated^5^, which is an important strategy for maintaining physiological homeostasis and acting on the central nervous system, including pain reduction^6^.

Some neurodegenerative diseases have an unknown etiology, but when it comes to diseases related to nerve synapses, the most studied systems include the GABAergic nervous system (GNS) and the CholNS^7,8^. The GNS comprises the most abundant synapses in the central region of the central nervous system, which are mediated by the action of the neurotransmitter γ-aminobutyric acid (GABA), and plays a dominant role in inhibitory processes^9^. The GABAergic neurotransmission occurs in interneurons that modulate local neurotransmission including noradrenergic, dopaminergic, and serotonergic neurons^10^. Thus, it is believed that the reduction of GABAergic transmission over a long period is associated with the various deleterious impairments of different neurotransmitters observed in Alzheimer's disease (AD)^10,11^. The CholNS is responsible for cognition, memory, and senses, and its neurotransmitter is acetylcholine (ACh). ACh is an excitatory neurotransmitter formed from acetyl coenzyme-A and choline, which acts at neuromuscular junctions, memory, and areas of the brain related to learning^12^. Thus, the CholNS acts in the peripheral nervous system along with the neuromuscular connections, being, therefore, affected in neurodegenerative diseases such as Parkinson's and Alzheimer's diseases^13^.

Changes that occur in the CholNS are also responsible for behavioral disturbances in AD patients^14,15^. In cholinergic signaling, the enzyme acetylcholinesterase (AChE) develops the nerve impulse through the hydrolysis of ACh into acetate and choline. Oh et al.^16^ showed that patients with AD enhanced when they were treated with AChE inhibitors. Similar investigations reported that acute CBD causes significant alterations in brain activity during relaxing conditions and performance of mental tasks in both healthy recruits and patients with a psychiatric illness^17^. The safety of CBD was also tested in *C. elegans* and no toxicity effects were observed^18^.

To understand neurological disorders and their effects on physiological processes, model organisms have been used. Among these, the free-living nematode *C. elegans* is consolidated in biochemical, toxicological, and genetic studies. The nematode *C. elegans* has different neurotransmitter systems that coordinate its behavior, including dopaminergic, cholinergic, serotonergic, glutamatergic, and GABAergic systems^19^. In addition, the *C. elegans* mutant strain *muscle:Abeta1-42* has the addition of the gene that encodes the human β-amyloid protein 1–42 peptides in body wall muscle, which is transcriptionally activated by heat stress (25 °C)^20^. The β-amyloid protein is one of the main proteins involved in the onset and progression of AD^21^.

Based on these studies and the gap in the potential of *C. sativa*-based CBD and THC oils, we hypothesized that these oils are effective in decreasing or delaying the symptoms caused by AD. This study aimed to evaluate the protective potential of different concentrations of CBD and THC cannabinoids obtained from *C. sativa* on behavioral parameters (pharyngeal pumping rate, defecation cycle, and the locomotor parameter of body bend), cholinergic system (AChE activity), and reactive oxygen species (ROS) in *C. elegans* wild-type and two transgenic strains, a *muscle:Abeta1-42* strain, that encodes the human β-amyloid 1–42 peptide in body wall muscle, and *muscle:Abeta-control* used as a control of *muscle:Abeta1-42*.

## Results

### The effect of *Cannabis sativa* oils exposure on *C. elegans* N2 wild-type

Here was evaluated the effect of the *C. sativa* oils on wild-type (N2) non-stressed *C. elegans* aiming to understand the effect of the oils applied alone in a healthy organism to observe the changes in the behavior (body bends, pharyngeal pumping, and defecation cycle) as well as on biochemical biomarkers such as acetylcholinesterase enzyme activity and ROS levels. In addition, evaluating in mutant worms the fluorescent antioxidant enzymes expression such as superoxide dismutase, catalase, glutathione-S-transferase, and heat shock protein even without stress.

**Figure 1:**
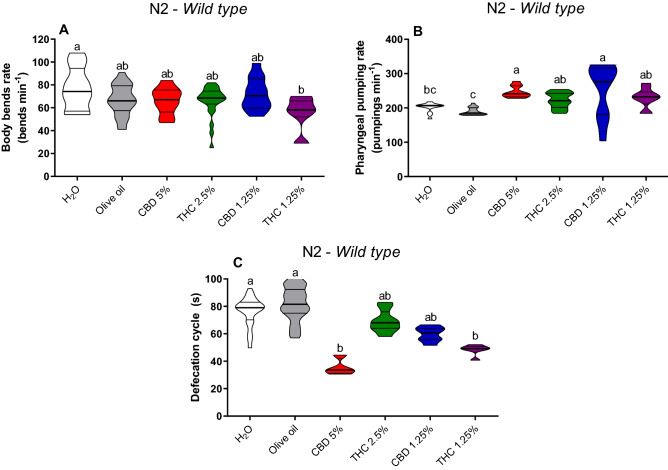
Body bends rate (**A**), pharyngeal pumping rate (**B**), and defecation cycle length (**C**) in *C. elegans* wild-type strain (N2). Data are expressed by the mean ± standard error (SEM). Means followed by the same letter are not significantly different according to Tukey’s test (*p* > 0.05) (**A**,**C**) or Dunn’s test (*p* > 0.05) (**B**).

On body bends rate was decreased in the wild-type (N2) strain after the 1.25% THC-treated group (*p* = 0.008) in comparison to the control group (Fig. 1A). The pharyngeal pumping on N2 strain was increased in the groups treated with CBD 1.25% and CBD 5% in comparison to water (*p* = 0.004; *p* = 0.02) and olive oil (*p* = 0.004; *p* = 0.0001) controls (Fig. 1B). Defecation cycle on N2 was decreased in the groups treated with CBD 5% and THC 1.25% in comparison to the water (*p* = 0.0001 and *p* = 0.004, respectively) and olive oil (*p* = 0.0001 for both) controls (Fig. 1C).

**Figure 2:**
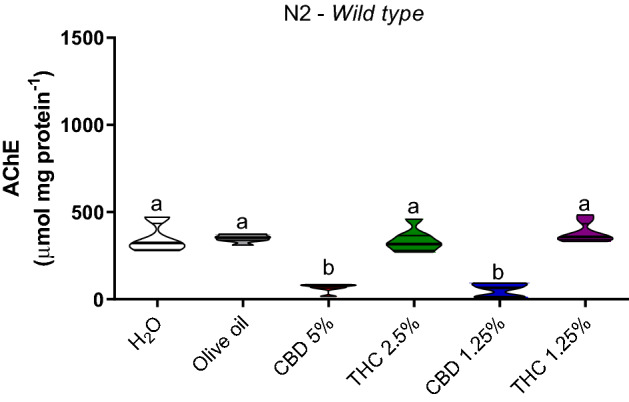
Acetylcholinesterase (AChE) activity in *C. elegans* wild-type strain (N2). Data are expressed by the mean ± standard error (SEM). Means followed by the same letter are not significantly different according to Tukey’s test (*p* > 0.05).

Acetylcholinesterase activity on N2 was decreased in both CDB-treated groups (1.25% and 5%) in comparison to the groups treated with water (*p* = 0.0001), olive oil (*p* = 0.0001), THC 2.5% (*p* = 0.0001), and THC 1.25% (*p* = 0.0001) (Fig. 2).

**Figure 3:**
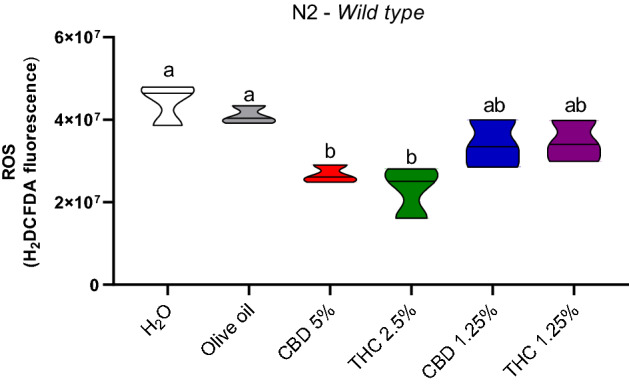
Reactive oxygen species (ROS) identification (H_2_DCFDA) in *C. elegans* wild-type strain (N2). Data are expressed by the mean ± standard error (SEM). Means followed by the same letter are not significantly different according to Tukey’s test (*p* > 0.05).

The ROS levels on N2 strain decreased in the groups treated with CBD 5% and THC 2.5% in comparison to water (*p* = 0.006; *p* = 0.001) and olive (*p* = 0.02; *p* = 0.005) controls, CBD 1.25% (*p* = 0.05; *p* = 0.04) and THC 1.25% (*p* = 0.05; *p* = 0.04) (Fig. 3).

**Figure 4:**
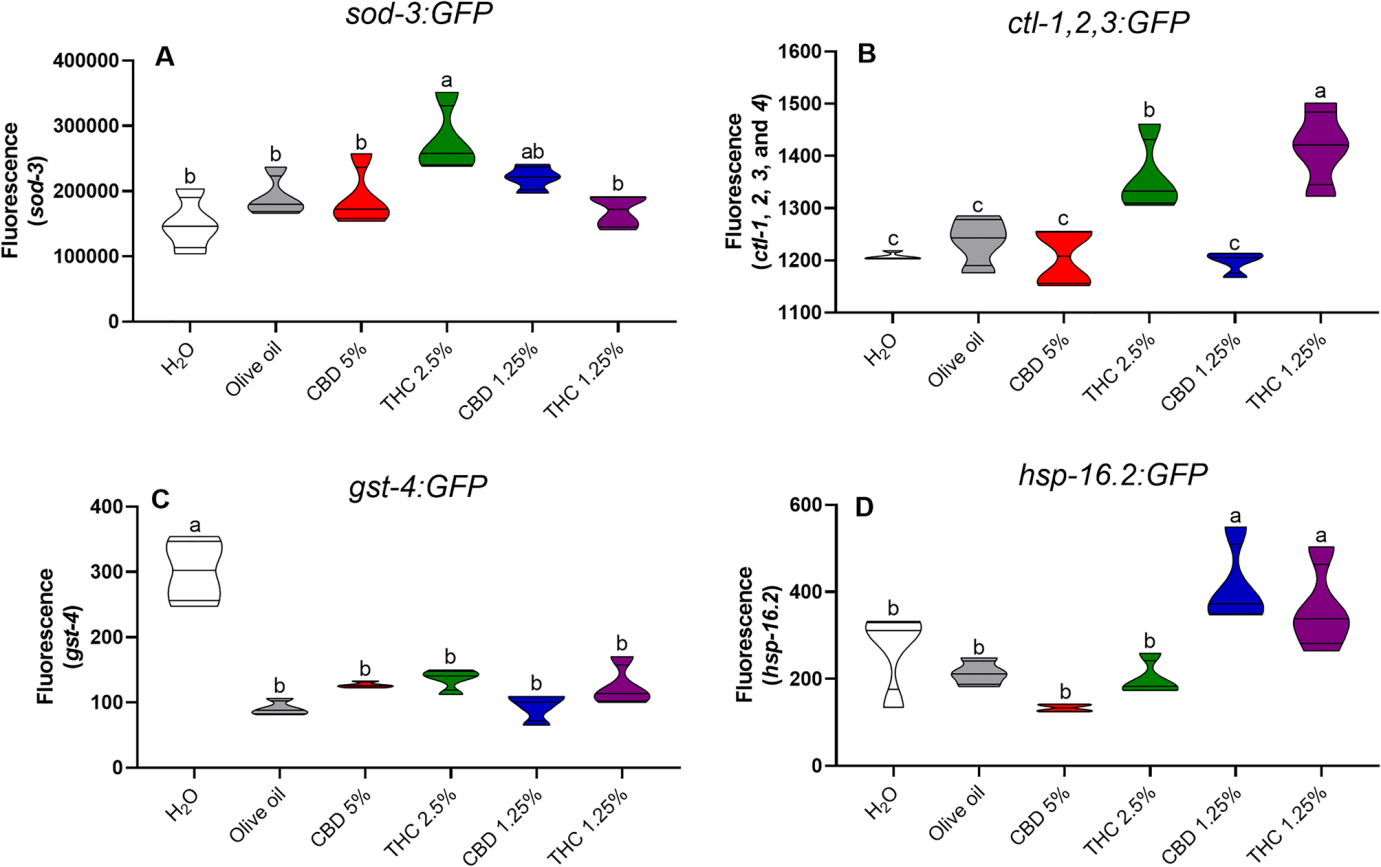
Antioxidant enzymes expression by green fluorescent protein (GFP) in *C. elegans Sod-3:GFP* mutant strain expressing superoxide dismutase (**A**), *Cat-1,2,3:GFP* mutant strain expressing catalase (**B**), *Gst-4:GFP* mutant strain expressing glutathione-S-transferase (**C**), and *Hsp-16.2:GFP* mutant strain expressing heat shock protein (**D**). Data are expressed by the mean ± standard error (SEM). Means followed by the same letter are not significantly different according to Tukey’s test (*p* > 0.05).

The SOD fluorescent expression in *C. elegans Sod-3:GFP* mutant strain was increased in the group treated with THC 2.5% in comparison to both water (*p* = 0.001) and olive oil (*p* = 0.04) controls, as well as CBD 5% (*p* = 0.04), and THC 1.25% (*p* = 0.008) (Fig. 4A). The CAT fluorescent expression in the *Cat-1,2,3:GFP* mutant strain increased in the group treated with THC 1.25% in comparison to water (*p* = 0.007) and olive oil (*p* = 0.0002) controls, CBD 5% (*p* = 0.0002), THC 2.5% (*p* = 0.004), CBD 1.25% (*p* = 0.0001) (Fig. 4B). In the *Gst-4:GFP* mutant strains, GST fluorescent expression decreased in comparison to all the treatment groups (*p* = 0.0001) (Fig. 4C). The HSP expression, in the *Hsp-16.2:GFP* mutant strain, was increased in the groups treated with CBD 1.25% and THC 1.25% in comparison to water (*p* = 0.01; *p* = 0.05) and olive oil (*p* = 0.03; *p* = 0.01) controls, as well as CBD 5% (*p* = 0.0004; *p* = 0.003) and THC 2.5% (*p* = 0.006; *p* = 0.04) treatments (Fig. 4D).

### Effect of *Cannabis* oils on strains *muscle:Abeta1-42* and *muscle:Abeta-control* heat-stressed and non-stressed

Here was evaluated the effect of the *C. sativa* oils on mutant strains that expresses β-amyloid aggregates on muscles (*muscle:Abeta1-*42 and its control strain *muscle:Abeta-control*) under heat stress as an indicator of Alzheimer’s condition in humans. For this, the worms were heat stressed and treated with the *C. sativa* oils and had the behavior (body bends, pharyngeal pumping, and defecation cycle) and biochemical (acetylcholinesterase activity) biomarkers evaluated.

**Figure 5:**
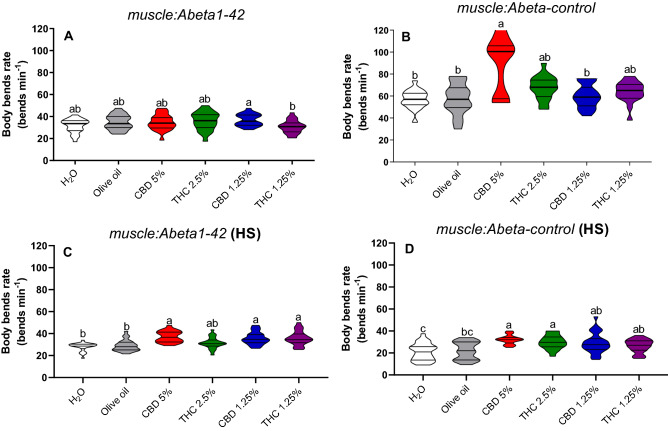
Body bends rate in non-stressed (**A**,**B**) and heat-stressed (HS) (**C**,**D**) *C. elegans muscle:Abeta1-42* and *muscle:Abeta-control* transgenic strains. Data are expressed by the mean ± standard error (SEM). Means followed by the same letter are not significantly different according to Tukey’s test (*p* > 0.05) (**A**,**C**) or Dunn’s test (*p* > 0.05) (**B**,**D**).

In the non-stressed worms on body bends, we had a decrease in the group treated with THC 1.25% (*p* = 0.01) in comparison to CBD 1.25% in the *muscle:Abeta1-42* transgenic strain (Fig. 5A). The body bends rate on *muscle:Abeta-control* increased in the group treated with CBD 5% in comparison to both water (*p* = 0.0006) and olive oil (*p* = 0.02) controls, as well as the group treated with CBD 1.25% (*p* = 0.008) (Fig. 5B).

On the other hand, body bends on heat-stressed (HS) strain *muscle:Abeta1-42* was increased in CBD 5% (*p* = 0.01), CBD 1.25%, and THC 1.25% in comparison to water (*p* = 0.001; *p* = 0.001; *p* = 0.001) and olive oil (*p* = 0.01; *p* = 0.01; *p* = 0.01) control treatments (Fig. 5C). In the HS *muscle:Abeta-control*, the body bends rate was increased in the groups treated with CBD 5%, and THC 2.5% in comparison to water (*p* = 0.0001), olive oil (*p* = 0.0001), CBD 1.25% (*p* = 0.0002; *p* = 0.0001) and THC 1.25% (*p* = 0.003; *p* = 0.04) (Fig. 5D).

**Figure 6:**
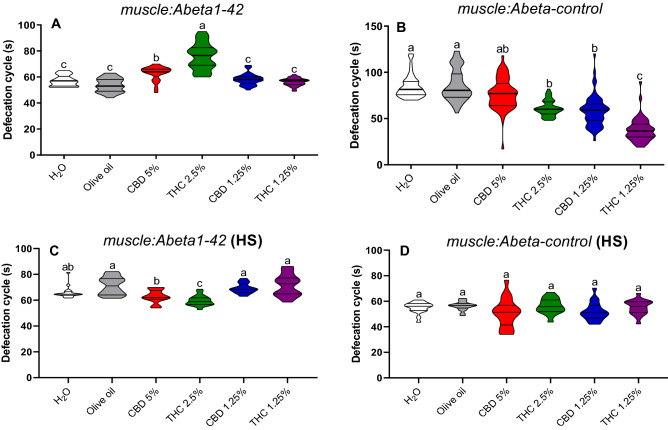
Defecation cycle rate in non-stressed (**A**,**B**) and heat-stressed (HS) (**C**,**D**) *C. elegans muscle:Abeta1-42* and *muscle:Abeta-control* transgenic strains. Data are expressed by the mean ± standard error (SEM). Means followed by the same letter are not significantly different according to Dunn’s test (*p* > 0.05).

Defecation cycle in *muscle:Abeta1-42* strain without stress, increased in the group treated with CBD 5% in comparison to the water (*p* = 0.001) and olive oil (*p* = 0.0001) controls, and also in comparison with CBD 1.25% (*p* = 0.02) and THC 1.25% (*p* = 0.0001) treatments (Fig. 6A). A similar response was observed in the defecation cycle in the group treated with THC 2.5% (*p* = 0.0001). Defecation cycle on *muscle:Abeta-control* strain was decreased in the groups treated with THC 2.5%, CBD 1.25%, and THC 1.25% in comparison to water (*p* = 0.0001) and olive oil (*p* = 0.0001) controls (Fig. 6B).

Defecation cycle on HS *muscle:Abeta1-42* decreased in the group treated with THC 2.5% in comparison to water (*p* = 0.04), olive oil (*p* = 0.0001), CBD 5% (*p* = 0.05), CBD 1.25% (*p* = 0.0001), and THC 1.25% (*p* = 0.0001) (Fig. 6C). The HS *muscle:Abeta-control* defecation cycle did not have significant changes between treatment groups (Fig. 6D).

**Figure 7:**
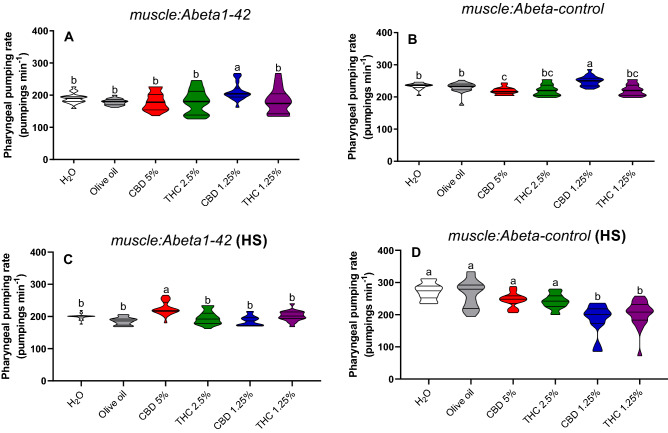
Pharyngeal pumping rate in non-stressed (**A**,**B**) and heat-stressed (HS) (**C**,**D**) *C. elegans muscle:Abeta1-42* and *muscle:Abeta-control* transgenic strains. Data are expressed by the mean ± standard error (SEM). Means followed by the same letter are not significantly different according to Tukey’s test (*p* > 0.05) (**B**) or Dunn’s test (*p* > 0.05) (**A**,**C**,**D**).

Without stress, the pharyngeal pumping increased in the *muscle:Abeta1-42* strain in the group treated with CBD 1.25% in comparison to the groups treated with water (*p* = 0.01), olive oil (*p* = 0.001), CBD 5% (*p* = 0.007), THC 2.5% (*p* = 0.01), and THC 1.25% (*p* = 0.02) (Fig. 7A). In the *muscle:Abeta-control* transgenic strain, the pharyngeal pumping was increased in the group treated with CBD 1.25% in comparison to water (*p* = 0.03), olive oil (*p* = 0.03), CBD 5% (*p* = 0.0001), THC 2.5% (*p* = 0.0001), and THC 1.25% (*p* = 0.0001) treatments (Fig. 7B). The pharyngeal pumping was decreased in the group treated with CBD 5% in comparison to water (*p* = 0.03), olive oil (*p* = 0.002), and CBD 1.25% (*p* = 0.0001).

Pharyngeal pumping on heat-stressed *muscle:Abeta1-42* was increased in the group treated with CBD 5% in comparison to the controls water (*p* = 0.006), olive oil (*p* = 0.0001), THC 2.5% (*p* = 0.0003), CBD 1.25% (*p* = 0.0001), and THC 1.25% (*p* = 0.02) treatments (Fig. 7C). On the heat-stressed *muscle:Abeta-control*, the pharyngeal pumping was decreased in the groups treated with CBD 1.25% and THC 1.25% in comparison to the water (*p* = 0.0001; *p* ≤ 0.0001), olive oil (*p* = 0.0001; *p* = 0.0005), CBD 5% (*p* = 0.001; *p* = 0.03), and THC 2.5% (*p* = 0.008; *p* = 0.007) (Fig. 7D).

### AChE activity in transgene strains that encodes the human β-amyloid 1–42 peptide in muscles (*muscle:Abeta1-42* and *muscle:Abeta-control*)

**Figure 8:**
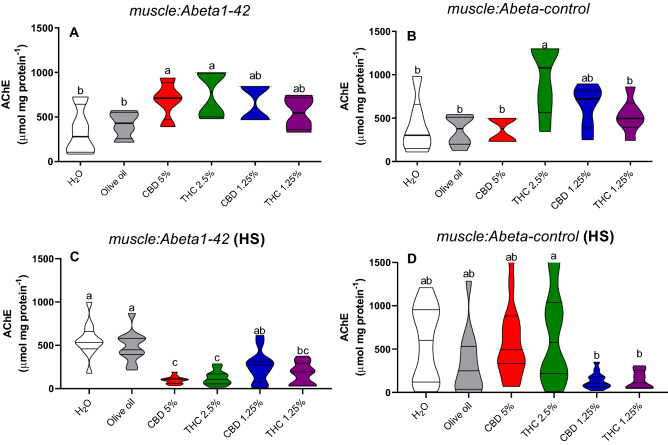
Acetylcholinesterase (AChE) activity in non-stressed (**A**,**B**) and heat-stressed (HS) (**C**,**D**) *C. elegans muscle:Abeta1-42* and *muscle:Abeta-control* transgenic strains. Data are expressed by the mean ± standard error (SEM). Means followed by the same letter are not significantly different according to Tukey’s test (*p* > 0.05) (**A**,**B**) or Dunn’s test (*p* > 0.05) (**C**,**D**).

AChE activity without stress was increased in *muscle:Abeta1-42* strain treated with CBD 5% and THC 2.5% in comparison to both water (*p* = 0.02; *p* = 0.004) and olive oil (*p* = 0.02; *p* = 0.05) controls (Fig. 8A). In the *muscle:Abeta-control* strain, AChE activity was increased in the group treated with THC 2.5% in comparison to both water (*p* = 0.01) and olive oil (*p* = 0.007) controls, as well as in the CBD 5% (*p* = 0.008), and THC 1.25% (*p* = 0.05) (Fig. 8B).

On heat-stressed *muscle:Abeta1-42*, AChE activity was decreased in the group treated with THC 1.25% in comparison to water (*p* = 0.007) and olive oil (*p* = 0.01) controls (Fig. 8C). In addition, the groups treated with CBD 5% and THC 2.5% had reduced activity in comparison to water (*p* = 0.0001; *p* = 0.0001) and olive oil (*p* = 0.0004; *p* = 0.001) controls as well as with the CBD 1.25% group (*p* = 0.01) (Fig. 8C). On the heat-stressed *muscle:Abeta-control* strain, the AChE activity was increased in the group treated with THC 2.5% in comparison to CBD 1.25% (*p* = 0.01) and THC 1.25% (Fig. 8D). In addition, the groups treated with CBD 1.25% and THC 1.25% had a reduced activity in comparison to water (*p* = 0.001), olive oil (*p* = 0.01), CBD 5% (*p* = 0.001), and THC 2.5% (*p* = 0.001) (Fig. 8D).

## Discussion

*Cannabis sativa*-based CBD and THC oils have shown potential benefits for *C. elegans* nematodes. First, when evaluating the wild-type N2 strain, an increase in pharyngeal pumping was observed when CBD oil was added. In addition, a reduction in the duration of the defecation cycle was observed after exposure to the highest dose of CBD and the lowest dose of THC, as well as the activity of the enzyme AChE. Both the pharynx and the defecation cycle are could be modulated by AChE levels in the synaptic clefts.

AChE is a central nervous system enzyme that acts in the synaptic cleft and neuromuscular junctions and it is responsible for the hydrolysis of acetylcholine (ACh) into acetic acid and choline^22^. The ACh, in turn, is one of the main neurotransmitters that make up the nervous system, being responsible for behavior, memory, movement, and reasoning^23^. Inhibition of AChE enzyme activity is responsible for causing high excitability in cholinergic neurons, in addition to forcing ACh to remain longer in the synaptic cleft, inducing an excitatory effect in the system. The reflexes of this excitatory effect are mostly evidenced in high muscle contraction, such as the increase in the frequency of pharyngeal pumping^24,25^. In nematodes, the pharynx plays an important role, as it is a bilobed neuromuscular organ where the pharyngeal ring is located, which contains the highest concentration of neurons in the individual^26^. As the pharynx is mainly regulated by the action of the ACh neurotransmitter, the increase in pharyngeal rhythm is related to an improvement in neurodegenerative diseases^27^.

The defecation cycle, resulting from muscle contractions, is also regulated by the ACh levels in the synaptic clefts. However, they are still strongly influenced by two other factors, namely calcium (Ca^+^) levels and the neurotransmitter γ-aminobutyric acid (GABA), which may indicate changes in the cholinergic and GABAergic systems due to a reduction in the time of the defecation cycle^24^. In addition, the accentuation of the reduction of the defecation interval is closely linked to the endocannabinoid system’s CB1 receptors, which are found throughout the central nervous system, peripheral, synaptic endings, intestine, and immune cells, being that in the intestine the endocannabinoid system plays an important role in its motility^28,29^. CB1 receptors act more actively in the presence of CBD, thus increasing the number of defective contractions and reducing the interval between them in *C. elegans*.

Still, in the wild-type N2 strain, the ROS levels were reduced after both CBD and THC exposure. And there was an increase in the activity of antioxidant enzymes, mainly SOD and CAT, and in HSP. These results show the potential of oils in reducing cellular damage and preventing lipid peroxidation, which could be harmful to cells.

ROS, also known as free radicals, are formed by oxidative stress in a substantial quantity. These free radicals or ROS are unstable due to the presence of unmatched electrons, these electrons could react with biomolecules (lipids, protein, nucleic acid, and carbohydrates), inducing irreversible damage^30^.

Moreover, in vitro studies exploring the impacts of cannabis extracts with various THC and CBD percentages on oxidative stress in differentiated neuronal SH-SY5Y cells using H_2_O_2_, demonstrate a considerable impact of high THC cannabis extracts to fight ROS formation^31^. However, CBD did not show as high an antioxidant action as THC under these test conditions^31^.

In the transgenic *muscle:Abeta1-42* strain that encodes the human β-amyloid 1–42 peptide in muscles, it was possible to observe that the highest CBD concentration increased worm movement and pharyngeal pumping, and reduced AChE activity in relation to the strain *muscle:Abeta-control*, used as a control. This indicates that for the *muscle:Abeta1-42* strain, where β-amyloid expression occurs, the release and/or action of ACh in the synaptic cleft needs to be more accentuated, so that correct signaling occurs, which demonstrates that *Cannabis*-based oils, especially CBD, could act as an aid in the process of improving cholinergic signaling in individuals with β-amyloid expression. Patients affected by Alzheimer's have aggregations of different isoforms of β-amyloid protein in the synaptic clefts, which under normal conditions would be precipitated and eliminated periodically^21^. These aggregations, in addition to hindering communication between neurons causing neurotransmitters to lose efficiency, are also responsible for the death of cholinergic neurons and thus leading to the progression of neurodegenerative diseases. In addition, in aging tests, a gradual decrease in the pharyngeal pumping is reported, which may be accelerated by neurodegenerative diseases^32^. Furthermore, the increase in the time between defecation cycles, related to the increase in AChE activity with the inhibition of the excitability of the cholinergic system, allows the reduction of the defecation cycle^20^.

Naturally, in *C. elegans* mutant strains for neurodegenerative diseases, as well as in patients affected by neurodegenerative diseases, there is a harmful decrease in the levels of neurotransmitters and also in the activity of enzymes that act in the synapse process^33^. To reverse this effect, defining compounds capable of activating and stabilizing these enzymes at normal levels is an important process to reduce the progression of neurodegenerative diseases. The modulation of enzymes such as AChE can positively influence neurotransmission in neurodegenerative patients since in many diseases such as Alzheimer's and Parkinson's, intense and dense protein neurofibrillary tangles are formed in the synaptic clefts. Therefore, the activation of nervous system enzymes intensifies the processes, equalizing the synapses^34^.

In addition, the endocannabinoid system receptors, mainly CB1, are important biomarkers of body weight gain. *Cannabis* is known to increases appetite, particularly in high-fat foods^35^. This occurs as the endocannabinoid system regulates energy balance and food intake at various functional levels, both in the brain and in the gastrointestinal tract^36^. Thus, the increase in the pharyngeal rate in the mutant strains can also be related to the food-inducing effect of these organisms. This is particularly important in patients suffering from neurodegenerative diseases with loss of appetite, where the use of oils can also favor an improvement in food intake.

Studies such as the one presented are important to clarify the true mechanisms by which fragmented or small improvements are observed in chronic diseases linked to the central nervous system. However, even more importantly, it is necessary to show through science, the need not discriminate between these therapies. Considering that, in many cases, it is the most effective and perhaps the only alternative available.

## Methods

### Obtaining the compounds

The oils were donated by APEPI—RJ (Support for Medicinal *Cannabis* Research and Patients), a non-profit initiative that aims to support research and also the individual cultivation of *Cannabis* for medicinal purposes. As the oils obtained were donated and the extraction protocols were not provided by APEPI, they were used for analysis on nematodes in the same way that they are marketed for medicinal purposes for patients with various diseases, including AD. The vehicle used was ultrapure olive oil, also received by the APEPI – RJ, and used as a control treatment.

### Analysis of major compounds from CBD and THC oils

Aliquots (100 mg) of crude CBD and THC oils were added to 10 mL of methanol:n-hexane solvent (9:1, v/v), homogenized in a vortex mixer for 1 min, and kept in an ultrasound bath for 30 min.

The samples were then refrigerated at − 20 °C for 30 min and centrifuged at 4000 rpm for 20 min. The supernatant was filtered through polytetrafluoroethylene membranes (PTFE, 0.22 µm) and stored in vials, in a refrigerator, for further analysis. For the analyses, a gas chromatograph coupled to the detector by mass spectrometry (GCMS 7890, Agilent Technologies, Wilmington, USA) was used with the following chromatographic conditions: column, DB5-HT (30 m × 250 µm × 0.1 µm); oven, 150 °C (1 min); 15 °C/min; 270 °C (6 min); 20 °C/min; 300 °C (2 min); running time: 18.5 min; flow: 0.6 mL/min; 1 µL injection, split: 50:1; injector and transfer line temperatures, 250 °C; mass spectrometer programmed for acquisition in Full Scan mode 50–500 m/z, electron ionization source (EI) 70 eV. The obtained mass spectra were compared to those from the Nist library.

Crude CBD oil contains 8.98% CBD and 1.22% THC, while crude THC oil contains 50.94% THC and 6.08% CBD.

### Caenorhabditis elegans

All the strains used in the study were obtained from the *Caenorhabditis* Genetics Center (CGC), University of Minnesota, Minneapolis, USA. The nematode strains were maintained in a nematode growth medium (NGM) with *Escherichia coli* OP50 at 20 °C^37^. The study was carried out in the Biochemistry Laboratory of the Federal Institute of Rio Grande do Sul, campus Sertão, Sertão, Brazil. All used strains are detailed in Table 1.

**Table 1:** List of strains used and insertion of the specific gene.

Strain name	Gene insertion	Gene expression	References
N2	–	Wild-type	Gems and Riddle^39^
*muscle:Abeta1-42* (GMC101)	dvIs100 [(Punc-54::A-beta(1–42)::unc-54 3Prime UTR; Pmtl2::GFP)]	A transgene strain that encodes the human β-amyloid 1–42 peptide in body wall muscle on 25 °C	Bortoli et al.^40^
*muscle:Abeta-control* (CL2122)	dvIs15 [(pPD30.38) unc-54(vector) + (pCL26) mtl-2::GFP]	Strain control of *muscle:Abeta1-42*	Bortoli et al. ^40^
*Cat-1,2,3:GFP* (GA800)	wuIs151 [ctl-1(+) + ctl-2(+) + ctl-3(+) + myo-2p::GFP]	Catalase green fluorescent expression (*ctl1*; *ctl2*; and *ctl3*)	Tamagno et al.^41^
*Gst-4:GFP* (CL2166)	dvIs19 [(pAF15)gst-4p::GFP::NLS]	Glutathione-S-transferase green fluorescent expression (*gst4*)	Tamagno et al.^41^
*Sod-3:GFP* (CF1553)	muIs84 [(pAD76) sod-3p::GFP + rol-6(su1006)]	Superoxide dismutase green fluorescent expression (*sod3*)	Tamagno et al.^41^
*Hsp-16.2:GFP* (CL2070)	dvIs70 [hsp-16.2p::GFP + rol-6(su1006)]	Heat shock protein green fluorescent expression (*hsp17.2*)	Tamagno et al.^41^

The transgenic strain *muscle:Abeta1-42* is widely used in DA. It encodes the human β-amyloid 1–42 peptide in body wall muscle^38^. The strain *muscle:Abeta1-42* had the induced expression of the protein β-amyloid through heat stress (25 °C), in larval stage L1. Strain *muscle:Abeta-control* is a negative control of strain *muscle:Abeta1-42*.

Different strains of *C. elegans* were also used to evaluate oxidative stress with the green fluorescent protein (GFP) assays: the strain CF1553, a mutant for superoxide dismutase (*sod-3* gene), GA800, a mutant for catalase (*ctl-1*, *2*, and *3*), CL2166, a mutant for glutathione-S-transferase (*gst-4* gene), and CL2070, a mutant used to determine the heat shock protein (*hsp-16.2*).

### Experimental conditions

For the non-stressed assays, the strains wild-type N2, *muscle:Abeta1-42* (GMC101), *muscle:Abeta-control* (CL2122), *Sod-3:GFP* (CF1553), *Gst-4:GFP* (CL2166), *Hsp-16.2:GFP* (CL2070), and *Cat-1,2,3:GFP* (GA800) were maintained at 20 °C. The nematodes were exposed to six different treatment groups: water control, olive oil control (used to prepare the CBD and THC oils), CBD 5%, THC 2.5%, CBD 1.25%, and THC 1.25%. They are exposed in the L4 stage.

The *C. elegans* nematodes were transplanted and sown in NGM, and kept in growth chambers at 20 °C, until they reached the L4 larval stage and after that they were synchronized (pregnant or with eggs)^42^. Nematodes feed with *E. coli*. At the L4 larval stage, the worms were washed 3 times, and centrifuged at 3000 rpm, for 2 min. Then, the nematodes were exposed to a synchronization in a 5 mL solution containing hypochlorite (1.5 ml) and sodium hydroxide (0.250 ml). The reagents break the cuticle and release their eggs. The eggs were placed on plates with the M9 buffer solution^36^, and the eggs were maintained at 20 °C, for approximately 24 h, until they reached the L1 larval stage. After that, they were transferred to NGM and fed with *E. coli*. On the next day, when they reach the L4 stage, they are able to be exposed to the treatments.

The nematodes were exposed to the treatments for 1 h. After that period, the nematodes were washed 3 times in M9 buffer solution, transferred to Petry dishes containing *E. coli* and acclimated for another 30 min for further behavioral evaluation.

For the assays with heat-stress (HS), the transgenic strains *muscle:Abeta1-42* and *muscle:Abeta-control* were raised to a temperature of 25 °C, after their synchronization. In this condition, the transgenic strain *muscle:Abeta1-42* encodes the human β-amyloid 1–42 peptide in body wall muscle. The exposition of the treatments was carried out with the same methods used for non-stressed nematodes.

### Behavioral assessment

Body bend rate, defecation cycles, and pharyngeal pumping rate were evaluated. All evaluations were performed in triplicate.

To evaluate the locomotor parameters of body bends, twenty-four nematodes were exposed to each treatment in the larval stage L4, where they were evaluated individually 2 times for 30 s, with a total time of 60 s for each nematode. These individuals were collected and placed in a Petry dish (5 cm diameter) containing agar for 1 min. Each of the nematodes was kept in a horizontal position (X axis) and the number of body curves generated in 30 s was counted. Body curvature was defined as a change in the direction of locomotion of the nematode's anterior part, i.e., the pharynx, along the Y axis, and expressed per minute^43^.

The duration of the defecation cycle was performed using a microscope (100 ×) (Primo Star, Zeiss, Germany). Twenty-four individuals, synchronously grown young adults, were examined per strain, per treatment. The time between two evacuative intervals was measured. Each nematode was observed for three consecutive defecation cycles and the time between the two consecutive cycles was calculated^43^.

To measure the pharyngeal pumping, twenty-four nematodes were observed three times for 10 s each, closing a total of 30 s (10 s-10 s-10 s)^44^. They are fed by sucking bacteria and grinding them in their terminal bulbs. As a complete cycle of synchronous contraction and relaxation of the terminal bulb is called a “pump”, the pharyngeal pumping rate (pumps per minute) was measured by visually observing the nematodes under a microscope (100×) (Primo Star, Zeiss, Germany).

### Acetylcholinesterase (AChE) enzyme activity

The AChE activity was determined in L4 stage nematodes, using a colorimetric assay^45^. After treatment exposure, 10.000 nematodes were washed three times with M9 buffer solution and transferred to microcentrifuge tubes. Samples were frozen and thawed 3 times in liquid nitrogen, followed by 5 rounds of sonication, each of 15 s in the ice, at 30% amplitude, with 10-s intervals between each. Then, the samples were centrifuged for 30 min at 15.000 rpm and the supernatants were collected. An aliquot (160 μL) of the supernatant was mixed with a solution containing DTNB (0.25 mM) and acetylthiocholine iodide (ASChI, 156 mM), and incubated at 30 °C for 5 min. The absorbance was measured in a spectrophotometer (UV-M51 Bel, Germany) at 405 nm, at 30 s-intervals, for 4 min. Kinetic measurements were recorded and converted to the total cholinesterase activity using the extinction coefficient (11.5 M^-1^ cm^−1^) of the 5-thio-2-nitro-benzoic acid. AChE activity was expressed as μmol ASChI min^−1^ mg^−1^ protein^46^.

### Antioxidant enzymes and heat shock protein

Determination of the green fluorescent expression of superoxide dismutase (SOD), catalase (CAT), glutathione-S-transferase (GST), and heat shock protein (HSP) was performed using a specific *C. elegans* mutant strain for each analysis. SOD activity was determined using the strain CF1552 [muls84 (pAD76) *sod-3p::*GFP + *rol-6(su1006*)], CAT with GA800 strain [wuls151 *ctl-1* + *ctl-2* + *ctl-3* + *myo-2*::GFP], GST with CL2166 strain [dvIs19 (pAF15) *gst-4p*::GFP::nls], and HSP with CL2070 strain (dvIs70 [hsp-16.2p::GFP + rol-6(su1006)]). These strains were exposed to the treatments as mentioned before. After that, the individuals were washed and transferred to 96-well microplates with M9 buffer solution, using excitation at 485 nm and emission at 530 nm to measure total fluorescence. Fluorescence-based assays were conducted on a microplate reader (Hidex, Chamelon, Chine), according to Tamagno et al.^41^ and Tamabara et al.^47^.

### Reactive oxygen species (ROS) levels

The ROS levels were evaluated in 1500 nematodes of the wild-type N2 strain. Nematodes in L4 stage were exposed to the six treatments, as previously described. After that period, they were washed out of treatment, and exposed to 2.7- dichlorofluorescein-diacetate (H_2_DCF-DA, 0.5 mM), for 1 h. The nematodes were washed again, containing the fluorescence dye, and then exposed to hydrogen peroxide (0.4 mM). The ROS levels were analyzed inside the nematodes by measuring fluorescence using excitation at 485 nm and emission at 535 nm, for 1 h in the presence of hydrogen peroxide. The results were expressed as function-time curves per mg of protein.

### Statistical analysis

The obtained data were submitted to previous analyses of homoscedasticity (Kolmogorov–Smirnov test) and homogeneity (Bartlett test). Parametric data were analyzed using a one-way analysis of variance (ANOVA), followed by Tukey's post hoc, and non-parametric data were compared using Dunn’s test. All analyses were evaluated at a 5% significant level. Statistical analysis was performed using Graph Pad Prism version software, version 8.0.1 (GraphPad Software, San Diego, USA).

## Data Availability

All data generated or analysed during this study are included in this published article. The raw datasets are available from the corresponding author on reasonable request.
